# Association of *Chlamydia trachomatis, C. pneumoniae*, and *IL-6* and *IL-8* Gene Alterations With Heart Diseases

**DOI:** 10.3389/fimmu.2019.00087

**Published:** 2019-02-05

**Authors:** Nubia Caroline Costa Almeida, Maria Alice Freitas Queiroz, Sandra Souza Lima, Igor Brasil Costa, Marco Antonio Ayin Fossa, Antonio Carlos R. Vallinoto, Marluísa de Oliveira Guimarães Ishak, Ricardo Ishak

**Affiliations:** ^1^Virus Laboratory, Institute of Biological Sciences, Federal University of Pará, Belém, Brazil; ^2^Virology Section, Evandro Chagas Institute, Ananindeua, Brazil; ^3^Cardiology Unit, Portuguese Beneficent Hospital, Belém, Brazil

**Keywords:** *Chlamydia pneumoniae*, *C. trachomatis*, polymorphisms, IL-6, IL-8, IL-10, TNF, CRP

## Abstract

Atherosclerosis is a progressive disease characterized by chronic inflammation of the arterial walls, associated with genetic and infectious factors. The present study investigated the involvement of *Chlamydia trachomatis* and *Chlamydia pneumoniae* infections and immunological markers (C-reactive protein, CRP, TNF-α, IL-6, IL-8, and IL-10) in the process of atherosclerosis. The evaluation included 159 patients for surgical revascularization (CAD) and 71 patients for surgical heart valve disease (HVD) at three hospitals in Belém, Brazil. The control group (CG) comprised 300 healthy individuals. Blood samples collected before surgery were used for antibodies detection (enzyme immunoassay), CRP (immunoturbidimetry) and IL-6 levels (enzyme immunoassay). Tissue fragments (atheroma plaque, heart valve and ascending aorta) were collected during surgery and subjected to qPCR for detection of bacterial DNA. Promoter region polymorphisms of each marker and relative quantification of *TNF-*α, *IL-8*, and *IL-10* gene expression were performed. Demography and social information were similar to the general population involved with both diseases. Antibody prevalence to *C. trachomatis* was 30.6, 20.3, and 36.7% (in the CAD, HVD, and CG, respectively) and to *C. pneumoniae* was 83.6, 84.5, and 80.3% (in the CAD, HVD, and CG, respectively). *C. trachomatis* cryptic plasmid DNA was detected in 7.4% of the samples. Frequency of *IL6*−174G>C polymorphism was higher in CAD and HVD than in CG regardless of previous exposure to *Chlamydia*. Previous *C. trachomatis* infection showed involvement in HVD and CAD. Significant association between disease and previous *C. pneumoniae* infection was found only among HVD. GG genotype of IL6−174G>C is apparently a risk factor for heart disease, whereas AT genotype of *IL8*−251A>T was mainly involved in valvulopathies, including patients with prior exposure to *C. pneumoniae*.

## Introduction

Atherosclerosis is a multifactorial condition that is influenced by genetic factors and lifestyle-related risk behaviors (1). Atherosclerosis is a progressive disease characterized by chronic inflammation of the arterial walls, which develops from the accumulation of low density lipoproteins in the arterial wall, oxidative, hemodynamic, biochemical, and inflammatory changes, and highly specific cellular responses (2, 3).

Local or systemic infections may contribute to the atherogenic process (4, 5). In particular, bacterial components of *Chlamydia pneumoniae* have been associated with atheroma plaque genesis and the potential risk of acute myocardial infarction (6, 7). The bacterium is capable of infecting smooth muscle cells, endothelial cells, and human macrophages (8–10). This association has been demonstrated through seroepidemiological, anatomic-pathological, and experimental studies and by demonstration of the presence of the bacteria in arterial walls (6, 7). *Chlamydia* infection in the arterial intima layer causes lesions that stimulate the inflammatory process (11, 12) by inducing elevated levels of immunological markers that influence the stability or progression of atherosclerotic plaques (11–13).

Although endothelial and cardiac muscle cells may not be common targets for *Chlamydia trachomatis* infection, this bacterium can reach the circulatory system through infected monocytes and macrophages. Infection of these cells occurs *in vitro* as evidenced by the presence of intracellular inclusions characteristic of bacterial multiplication (14). Analysis of *C. trachomatis* primary rRNA has also shown the existence of viable and metabolically active microorganisms inside the inclusions (15, 16).

*Chlamydia pneumoniae*-infected individuals have elevated plasma C-reactive protein (CRP) levels and atheroma plaques located in the carotid artery (17). Polymorphisms in the inflammatory and immune response genes lead to changes in the expression levels of these molecules (18, 19).

The present work investigated the association of immune response gene polymorphisms (*TNF-*308A>G, *IL-6*-174G>C, *IL-8*-251A>T, *IL-10-*1082G>A, and *CRP-*717T>C) and gene expression levels (mRNA and plasma levels) in patients with coronary disease associated with previous *C. pneumoniae* and *C. trachomatis* infections.

## Materials and Methods

### Study Population

A cross-sectional, case-control study was conducted with one group of 159 patients (109 men and 50 women) with coronary artery disease (CAD group) presenting with severe arterial obstruction with or without ischemia and a second group of 71 patients (30 men and 41 women) with heart valve disease (HVD group) presenting with a cardiac volume overload and high blood pressure. The patients with coronary disease had a surgical indication for myocardial revascularization, and the patients with valvulopathy had a surgical indication for valve prosthesis implantation (mitral or aortic).

The inclusion criteria included hospitalized individuals with an indication for one of the surgical procedures for the first time and the absence of antibiotic use. Exclusion criteria included individuals without an indication for surgery or indicated for repeat surgery those using antibiotics in the preoperative period. The samples were collected from November 2010 to July 2012 at the Portuguese Beneficent Hospital, the Ordem Terceira Hospital, and the Gaspar Viana Clinical Hospital Foundation, in the city of Belém, Pará state, Brazil.

A control group (CG) was formed with 300 individual blood donors (150 men and 150 women) from the Pará Hemotherapy and Hematology Foundation Center (HEMOPA) to compare the frequency of antibodies to *Chlamydia*, previous infection with the two species investigated, the frequency of immunological and inflammatory marker polymorphisms, and their gene expression levels (mRNA and plasma levels). The control group was matched with the cardiac patient groups (CAD and HVD) by sex and age and had no history and symptoms suggestive of heart disease.

The project was submitted and approved by the HEMOPA Human Research Ethics Committee (Protocol no. 0011.0.324.000–09). The subjects were informed about the project, and those who agreed to participate signed an informed consent form.

### Sample Collection and Storage

A sample of 10 mL of blood was collected by intravenous puncture using a vacuum collection system containing EDTA as the anticoagulant. The samples were processed for the separation of plasma and leukocytes, both of which were stored at −20°C prior to use. Plasma was used for the detection of specific antibodies to *C. pneumoniae* and *C. trachomatis* species and for analysis of the plasma CRP and interleukin 6 (IL-6) levels. Leukocytes were used for genomic DNA extraction to analyze polymorphisms and gene expression. The leukocyte samples used for the gene expression assays were stored in RNAlater® (Invitrogen, Carlsbad, CA, USA) at −70°C.

The following samples were collected from the patients who underwent surgical procedures: (i) fragments of the ascending aorta and when possible and at medical discretion coronary atheroma plaques (endarterectomy) during the revascularization procedure and (ii) mitral and aortic valve fragments during the valve replacement procedure. The samples were stored in tubes containing RNAlater® (Invitrogen, Carlsbad, CA, USA).

### Detection of Antibodies to *Chlamydia*

The plasma samples were tested by enzyme-linked immunosorbent assay (ELISA) to detect antibodies against *C. trachomatis* (NovaLisa™ *C. trachomatis* IgM and IgG) and *C. pneumoniae* (NovaLisa™ *C. pneumoniae* IgM and IgG) according to established protocols from the manufacturer (NovaTec, Dietzenbach, Offenbach, Germany).

Identification of the genetic polymorphisms *TNF-*308G>A (rs1800629), *IL6*-174G>C (rs1800795), *IL8*−251A>T (rs4073), *IL10*−1082G>A (rs1800896), and *CRP -*717T>C (rs2794521).

DNA was extracted from the peripheral blood leukocytes using the phenol-chloroform method. The procedure followed the cell lysis, protein precipitation, DNA precipitation, and DNA hydration steps. The obtained DNA was quantified using the Qubit® 2.0 Fluorometer (Life Technologies, Carlsbad, CA, USA) and the Qubit™ DNA Assay Kit solutions (Life Technologies, Carlsbad, CA, USA) following the protocol recommended by the manufacturer.

The *TNF -*308G>A, *IL6*−174G>C, and *CRP -*717T>C polymorphisms were assessed using polymerase chain reaction (PCR) followed by RFLP (restriction fragment length polymorphism) analysis. The reactions consisted of amplification of a 107-bp segment of the tumor necrosis factor (*TNF)* gene promoter region, a 169-bp segment of the *IL6* gene promoter region, and a 541-bp sequence of the *CRP* gene promoter region (20). For amplification of the *IL6* and *CRP* gene segments, specific primers were designed for each of the regions of interest using the Primer3 version 0.4.0 and FastPCR version 6.2 softwares based on the human reference sequences for the genes (NC_000001.11 and NC_000007.14, respectively). The reactions were conducted in an Eppendorf Mastercycler thermal cycler (Eppendorf, Hamburg, Germany) in a final 50-μL volume containing 100 ng of extracted total DNA, 200 nM each dNTP, 200 nM each primer (a pair specific for each gene), 1.5 mM MgCl_2_, 50 mM KCl, 10 mM Tris-HCl (pH 8.3), and 1.5 U of Taq DNA polymerase (Invitrogen, Carlsbad, CA, USA).

The sequences of the primers used for partial amplification of the *TNF* gene were TNFA-F: 5′-AGGCAATAGGTTTTGAGGGCCAT-3′ and TNFA-R: 5′-TCCTCCCTGCTCCGATTCCG-3′. The following temperature and cycling protocol was used: initial denaturation at 95°C for 5 min, 35 cycles of 30 s at 94°C (denaturation), 30 s at 53°C (hybridization), and 1 min at 72°C (extension), and a final extension of 10 min at 72°C. Amplification of the *IL6* gene segment utilized the primer pair IL6-f: 5′-TTGTCAAGACATGCCAAGTGCT-3′ and IL6-r: 5′-GCCTCAGAGACATCTCCAGTCC-3′. The temperature and cycling conditions were as follows: initial denaturation at 95°C for 5 min; 35 cycles of 30 s at 95°C (denaturation), 45 s at 59°C (hybridization), and 45 s at 72°C (extension); and a final extension of 10 min at 72°C. For the *CRP* gene segment, the primer pair CRP-f: 5′-ACTGGACTTTTACTGTCAGGGC-3′ and CRP-r: 5′-ATTCCCATCTATGAGTGAGAACCT-3′ was used. The temperature and cycling protocol was as follows: initial denaturation at 95°C for 5 min; 40 cycles of 30 s at 95°C (denaturation), 40 s at 60°C (hybridization), and 1 min at 72°C (extension); and a final extension of 10 min at 72 °C.

The RFLP protocol used the *NCO*I, *Nla*III, and *BstU*I enzymes (Invitrogen, Carlsbad, CA, USA) to genotype *TNF -*308G>A, *IL6*−174G>C and *CRP -*717T>C, respectively. The *TNF -*308G>A genotypes were identified by the presence of the 107-bp (AA) and 87-bp + 20-bp (GG) fragments. For *IL-6*-17G>C, the genotypes were identified by fragments of 169 bp (GG) and 87 bp + 80 bp (CC). The *CRP -*717T>C genotypes were identified by the presence of 541-bp (CC) and 297-bp + 244-bp (TT) fragments. The fragments were visualized after electrophoresis (100 V, 45 min) of the amplification product on a 3% agarose gel in 1x TAE buffer (TAE 40x stock – 1.6 M Tris base, 0.8 M sodium acetate, and 10 mg/mL of EDTA) using the SYBR^®;^ Safe DNA gel stain (Invitrogen, Carlsbad, CA, USA) and a UV transilluminator (Kasvi, Curitiba, Paraná, Brazil).

Polymorphisms in the *IL8* and *IL10* genes were identified by real-time PCR (qPCR) using TaqMan-SNP Genotyping Assays (Applied Biosystems, Foster City, CA, USA). The polymorphisms *IL8*−251T>A (rs4073) and *IL10*−1082A>G (rs1800896) were identified using the C_11748116_10 and C_1747360_10 assays, respectively. The reactions occurred in the StepOnePlus™ Real-Time PCR System (Applied Biosystems, Foster City, CA, USA) following the manufacturer's guidelines for an initial incubation of 10 min at 95°C and 40 cycles of 15 s at 95°C and 1 min at 60°C.

### *TNF, IL8*, and *IL10* Gene Expression

Total RNA was extracted from peripheral blood leukocytes using the Total RNA Purification Kit (Norgen Biotek Corporation, Thorold, ON, Canada). The concentration of the extracted RNA (ng/μL) was obtained using the Qubit® 2.0 fluorometer (Invitrogen, Carlsbad, CA, USA) according to the manufacturer's specifications, and the RNA integrity was examined using a 1% agarose gel stained with ethidium bromide. After the concentration and integrity check, the total RNA samples were normalized to 60 ng/μL for cDNA synthesis using the High Capacity cDNA Reverse Transcription kit (Applied Biosystems, Foster City, CA, USA). The cDNA was subjected to qPCR for relative quantification of the indicated markers with the Taqman® Gene Expression Assays Hs00174128_m1 (*TNF*), Hs00174103_m1 (*IL8*), and Hs00174086_m1 (*IL10*) in the StepOnePlus Sequence Detector (Applied Biosystems, Foster City, CA, USA) using the *GAPDH* gene as the internal control. The expression levels were calculated using the comparative CT method. For the relative quantification of *TNF* gene expression, 22 samples from patients with CAD, 17 samples from patients with HVD, and 28 samples from the GC were included. For *IL8* expression, 27 samples from patients with CAD, 21 samples from patients with HVD, and 32 samples from the CG were used. For the *IL-10* expression analysis, 27 samples from the patients with CAD, 20 samples from the patients with HVD, and 33 samples from the GC were tested.

### Plasma CRP and IL-6 Levels

The CRP levels were measured by immunoturbidimetry using the DiaSys® PCR U-hs kit (DiaSys, Waterbury, CT, USA) on the Architect c8000/Abbott® automated system (Abbott Park, Chicago, IL, USA) in samples from 159 people in the CAD group, 71 in the HVD group, and 196 in the CG group. The IL-6 levels were measured in samples from 19 people in the CAD group, 14 in the HVD group, and 28 CG subjects. Samples were tested in duplicate using the Novex ELISA Human IL-6 kit (Thermo Fisher Scientific, Waltham, MA, USA) following the manufacturer's instructions. The samples used for the IL-6 measurement were randomly selected using the BioEstat 5.3 software.

### qPCR to Identify *C. pneumoniae* and *C. trachomatis*

DNA was extracted from the aorta, valve, and atheroma plaque samples obtained during surgery using the QIAamp DNA Mini Kit (QIAGEN, Hilden, Mettmann, Germany). The presence of *C. pneumoniae* and *C. trachomatis* was investigated with qPCR using the *C. trachomatis* Q-PCR Alert Kit and the *C. pneumoniae* Q-PCR Alert Kit (Nanogen Advanced Diagnostic, Trezzano Sul Naviglio, Lombardy, Italy). For the *C. trachomatis* detection kit, a specific amplification reaction was performed for the cryptic plasmid (~7–10 copies/cell). *C. pneumoniae* was identified by amplification of a region of the *ompA* gene. A positive control for each species, a negative control, and an internal control for the β-globin gene were included in all reaction sets. The reactions were performed in a StepOnePlus Sequence Detector (PE Applied Biosystems, Foster City, CA, USA).

### Statistical Analysis

The information obtained through the epidemiological questionnaire was inserted into a database created in Microsoft Access version 2007. The serological results among the different groups were compared using the Chi-square test (χ^2^). The Hardy-Weinberg equilibrium was calculated to evaluate the genotype frequency distributions. The genotypic and allelic frequencies were calculated by direct counting, and differences between groups were evaluated using the χ^2^ and G-tests.

The associations of the plasma gene expression levels of pro- and anti-inflammatory markers with the presence of antibodies to *C. trachomatis* and *C. pneumoniae* were analyzed using the non-parametric Kruskall–Wallis and Mann–Whitney tests. The statistical analysis was performed using the GraphPad Prism version 5.0 and BioEstat 5.3 softwares. Significant associations were considered to have a *p* < 0.05.

## Results

### Demographic Information, Presence of Antibodies, and Bacterial DNA Detection

The demographic information (Table 1) showed that the majority of the patients were men in the CAD group (68.5%) and were women in the HVD group (57.7%). The ages ranged from 36 to 79 years (mean of 60.4 years) in the CAD group and from 14 to 80 years (mean of 45.6 years) in the HVD group. The majority of the CAD group patients were married (69.9%), and the greater frequency of the HVD patients were single (44.9%). In both groups, the most common education level was ≤ 5 years (79% in CAD and 68.6% in HVD), and the most common family income was ≤ 3 times the minimum wage (85.5 and 98.6%, respectively). The control group consisted of people aged between 24 and 68 years (mean age 40.3 years).

**Table 1:** Demographic and social characteristics of cardiac patients (CAD and HVD) investigated.

**Demographic and social characteristics**		**CAD *N* = 159 *N* (%)**	**HVD *N* = 71 *N* (%)**	**CG *N* = 300 *N* (%)**
Gender	Male	109 (68.5)	30 (42.3)	150 (50)
	Female	50 (31.5)	41 (57.7)	150 (50)
Age	Change (years)	36–79	14–80	24–68
	Average (years)	60.4	45.6	40.3
Marital status	Single	20 (13.1)	31 (44.9)	–
	Married	107 (69.9)	28 (40.6)	–
	Divorced/Widowed	26 (17)	10 (14.5)	–
	No information	6	2	–
Years of education	0	10 (6.4)	7 (10)	–
	< 2	16 (10.2)	3 (4.3)	–
	2– < 5	78 (49.7)	34 (48.6)	–
	5	20 (12.7)	4 (5.7)	–
	< 12	10 (6.4)	9 (12.9)	–
	12–16	23 (14.6)	13 (18.5)	–
	No information	2	1	–
Family income relative to minimum wage	< 1 x	22 (14.5)	18 (26.1)	–
	1 to 3 x	108 (71)	50 (72.5)	–
	≥4 x	22 (14.5)	1 (1.4)	–
	No information	7	2	–

CAD, coronary artery disease group; HVD, heart valve disease group; CG, control group

*N, number of individuals*.

Antibodies (Table 2) for *C. trachomatis* were detected in 30.6% (48/157) of the patients in the CAD group and 20.3% (14/69) of the patients in the HVD group. In the CG, the prevalence was 36.7% (103/281), which was significantly higher than the prevalence in the HVD group (*p* = 0.014). The prevalence rates of antibodies to *C. pneumoniae* in the three groups were 83.6% (133/159), 84.5% (60/71), and 80.3% (237/295), respectively, with no significant differences between groups. All undetermined serology results were excluded from the statistical tests.

**Table 2:** Frequency of antibodies to *C. trachomatis* and *C. pneumoniae* among cardiac patients (CAD and HVD) investigated.

**Previous infection**	**Presence of antibodies (IFI)**	**CAD *N* = 159 *N* (%)**	**HVD *N* = 71 *N* (%)**	**CG *N* = 300 *N* (%)**	***p*1**	***p*2**	***p*3**
*C. trachomatis*	Positive	48 (30.6)^a^	14 (20.3)^b^	103 (36.7)^c^	0.2383	0.014	0.1516
	Negative	109 (69.4)	55 (79.7)	178 (63.3)			
*C. pneumoniae*	Positive	133 (83.6)	60 (84.5)	237 (80.3)^e^	0.4596	0.5240	0.9757
	Negative	26 (16.4)	11 (15.5)	58 (19.7)			

*CAD, coronary artery disease group; HVD, heart valve disease group; CG, control group*.

p1: CAD vs. CG; p2: HVD vs. CG; p3: CAD vs. HVD.

a *2 samples not included; n = 157*.

b *2 samples not included; n = 69*.

c *19 samples not included; n = 281*.

e *5 samples not included; n = 295*.

Bacterial DNA from the two species was investigated in 81 samples (atheroma plaque, valve, and aorta). The cryptic plasmid DNA of *C. trachomatis* was detected in six (7.4%) samples; four of these samples were derived from aorta fragments and two from the mitral valve (Table 3). No DNA was detected in the atheroma plaques. Two samples were positive for anti-*C*. *trachomatis* IgM. The quantification of the risk measured by the CRP level showed that three samples showed a high risk of heart disease.

**Table 3:** Serology results for *C. trachomatis* according to the patient group, the origin of the tissues investigated for the presence of the cryptic plasmid and CRP expression.

**Group record**	**Sample**	**Anti-*****C. trachomatis***		**qPCR**	**CRP (mg/dL)**	**Risk**
		**IgG**	**IgM**			
22823 CAD	Aorta	+	–	+	0.17	No
22944 CAD	Aorta	+	–	+	0.12	No
22814 CAD	Aorta	+	–	+	0.66	Yes
22990 CAD	Aorta	-	+	+	0.39	Yes
23113 HVD	Mitral V	+	–	+	0.12	No
22988 HVD	Mitral V	_	+	+	1.69	Yes

*CAD, coronary artery disease group; HVD, heart valve disease group; CG, control group*.

*N, number of individuals*.

*qPCR, real-time PCR*.

*CRP, C-reactive protein*.

*Risk, risk of developing atherosclerosis based on the CRP levels*.

### Genetic Polymorphisms

The distribution of the polymorphisms of *CRP, TNF*, and *IL10* genes showed no significant changes irrespective of a previous infection or not with *Chlamydia*.

The genotypic distribution of the *IL-6*-174G>C polymorphisms (Table 4) showed a higher frequency of GG in the diseased groups (CAD and HVD) than in the controls (*p* = 0.0002). The frequency distribution according to previous exposure to the genus *Chlamydia* showed similar differences but significantly higher when compared to the controls without antibodies (*p* = 0.0158 and 0.0231 for the HVD and CAD, respectively) and with antibodies (*p* = 0.0003 and 0.0051, respectively). The presence of markers of previous exposure to *C. trachomatis* showed a significant difference between HVD and CG and between CAD and HVD (*p* = 0.0199 and 0.0122, respectively). A difference was found only between the HVD and control groups when the comparison was performed according to previous exposure to *C. pneumoniae* (*p* = 0.0368). The frequency of the ^*^G allele was usually higher in the CAD group, sometimes significantly, than the frequency of the ^*^C allele.

**Table 4:** Genotypic and allelic distributions of *IL-6*-174G>C markers among cardiac patients according to the presence of antibodies to *Chlamydia* and to the *C. trachomatis* and *C. pneumoniae* species.

***IL-6*-174G>C**	**Groups investigated**			***p*1**	***p*2**	***p*3**
	***N* (%)**	***N* (%)**	***N* (%)**			
	**CAD (*N* = 159)**	**HVD (*N* = 71)**	**CG (*N* = 300)**			
GG	134 (84.28)	60 (84.51)	207 (69.00)	0.0020^†^	0.0002^†^	0.7616^†^
GC	19 (11.95)	07 (9.86)	85 (29.33)			
CC	06 (3.77)	04 (5.63)	08 (2.67)			
^*^G	0.90	0.89	0.83	0.0049^†^	0.0852^†^	0.9196^†^
^*^C	0.10	0.11	0.17			
	**CAD (*****N*** **= 138)** ***Chlamydia+***	**HVD (*****N*** **= 61)** ***Chlamydia+***	**CG (*****N*** **= 51)** ***Chlamydia–***			
GG	118 (85.51)	51 (83.61)	35 (68.63)	0.0158^†^	0.0231^†^	0.6778^†^
GC	15 (10.87)	06 (9.83)	15 (29.41)			
CC	05 (3.62)	04 (6.56)	01 (1.96)			
^*^G	0.91	0.88	0.83	0.0568^*^	0.3543^*^	0.5720^*^
^*^C	0.09	0.12	0.17		
	**CAD (*****N*** **= 138)** ***Chlamydia+***	**HVD (*****N*** **= 61)** ***Chlamydia+***	**CG (*****N*** **= 247)** ***Chlamydia+***			
GG	118 (85.51)	51 (83.61)	171 (69.23)	0.0003^†^	0.0051^†^	0.6778^†^
GC	15 (10.87)	06 (9.83)	69 (27.94)			
CC	05 (3.62)	04 (6.56)	07 (2.83)			
^*^G	0.91	0.88	0.83	0.0042^*^	0.1910^*^	0.5720^*^
^*^C	0.09	0.12	0.17			
	**CAD (*****N*** **= 48)** ***C. trachomatis+***	**HVD (*****N*** **= 14)** ***C. trachomatis+***	**CG (*****N*** **= 178)** ***C. trachomatis–***			
GG	43 (89.58)	9 (64.29)	122 (68.54)	0.6517^†^	0.0199^†^	0.0122^†^
GC	03 (6.25)	03 (21.43)	51 (28.65)			
CC	02 (4.17)	02 (14.28)	05 (2.81)			
^*^G	0.93	0.75	0.83	0,0255^†^	0.4433^†^	0.0350^†^
^*^C	0.07	0.25	0.17			
	**CAD (*****N*** **= 133)** ***C. pneumoniae+***	**HVD (*****N*** **= 60)** ***C. pneumoniae+***	**CG (*****N*** **= 58)** ***C. pneumoniae–***			
GG	113 (84.96)	51 (85.00)	41 (70.69)	0.0561^†^	0.0368^†^	0.6020^†^
GC	15 (11.28)	05 (8.33)	15 (25.86)			
CC	05 (3.76)	04 (6.67)	02 (3.34)			
^*^G	0.91	0.89	0.84	0.0554^*^	0.2919^*^	0.7095^*^
^*^C	0.09	0.11	0.16			

*N, number of individuals*.

* *Chi-square test*.

† *G test*.

*p1, CAD vs. CG; p2, HVD vs. CG; p3, CAD vs. HVD*.

The AT genotype distribution of the *IL8*-251A>T polymorphisms (Table 5) was significantly different between the HVD and control groups (*p* = 0.0331), and the AT and AA genotypes differed between the diseased groups (*p* = 0.0367). A difference was also found when the presence of antibodies to *Chlamydia* species was compared to the controls without antibodies (*p* = 0.0179) and with antibodies (*p* = 0.0179). Previous exposure to *C. pneumoniae* showed involvement of the AT and TT genotypes, which significantly differed between the HVD and control groups (*p* = 0.0198) and between both diseased groups (*p* = 0.0172), but not with previous exposure to *C. trachomatis*. No differences were observed among the allele frequencies of the polymorphisms.

**Table 5:** Genotypic and allelic distribution of *IL-8-*251A>T markers among cardiac patients according to the presence of antibodies to *Chlamydia* and to the *C. trachomatis* and *C. pneumoniae* species.

**IL-8-251A>T**	**Groups investigated**			***p*1**	***p*2**	***p*3**
	***N* (%)**	***N* (%)**	***N* (%)**			
	**CAD (*N* = 159)**	**HVD (*N* = 71)**	**CG (*N* = 300)**			
TT	59 (37.11)	27 (38.03)	96 (32.00)	0.4914^*^	0.0331^*^	0.0367^*^
AT	76 (47.80)	24 (33.80)	150 (50.00)			
AA	24 (15.01)	20 (28.17)	54 (18.00)			
^*^T	0.61	0.55	0.57	0.2707^*^	0.7237^*^	0.2618^*^
^*^A	0.39	0.45	0.43			
	**CAD (*****N*** **= 138)** ***Chlamydia+***	**HVD (*****N*** **= 61)** ***Chlamydia+***	**CG (*****N*** **= 51)** ***Chlamydia–***			
TT	48 (34.78)	24 (39.34)	18 (35.29)	0.9676^*^	0.0513^*^	0.0179^*^
AT	69 (50.00)	19 (31.15)	26 (50.98)			
AA	21 (15.22)	18 (29.51)	7 (13.73)			
^*^T	0.61	0.55	0.57	0.9536^*^	0.4539^*^	0.4253^*^
^*^A	0.39	0.45	0.43			
	**CAD (*****N*** **= 138)** ***Chlamydia+***	**HVD (*****N*** **= 61)** ***Chlamydia+***	**CG (*****N*** **= 247)** ***Chlamydia+***			
TT	48 (34.78)	24 (39.34)	77 (31.17)	0.5816^*^	0.0269^*^	0.0179^*^
AT	69 (50.00)	19 (31.15) (-)	123 (49.80)			
AA	21 (15.22)	18 (29.51) (+)	47 (19.03)			
^*^T	0.60	0.55	0.56	0.3564^*^	0.8980^*^	0.4253^*^
^*^A	0.40	0.45	0.44			
	**CAD (*****N*** **= 48)** ***C. trachomatis+***	**HVD (*****N*** **= 14)** ***C. trachomatis+***	**CG (*****N*** **= 178)** ***C. trachomatis-***			
TT	16 (33.3)	5 (35.71)	57 (32.02)	0.9176^†^	0.9494^†^	0.9293^†^
AT	23 (47.92)	7 (50.00)	91 (51.12)			
AA	9 (18.75)	2 (14.29)	30 (16.85)			
^*^T	0.57	0.61	0.58	0.9483^*^	0.9012^*^	0.9161^*^
^*^A	0.43	0.39	0.42			
	**CAD (*****N*** **= 133)** ***C. pneumoniae+***	**HVD (*****N*** **= 60)** ***C. pneumoniae+***	**CG (*****N*** **= 58)** ***C. pneumoniae-***			
TT	46 (34.59)	24 (40.00)	19 (32.76)	0.8787^†^	0.0198^†^	0.0172^*^
AT	66 (49.62)	18 (30.00) (-)	31 (53.45)			
AA	21 (15.79)	18 (30.00)(+)	8 (13.79)			
^*^T	0.60	0.55	0.60	0.9220^*^	0.5726^*^	0.4845^*^
^*^A	0.40	0.45	0.40			

*N, number of individuals*.

* *Chi-square test*.

† *G test*.

*p1, CAD vs. CG; p2, HVD vs. CG; p3, CAD vs. HVD*.

### Plasma CRP and IL-6 Levels

**Figure 1:**
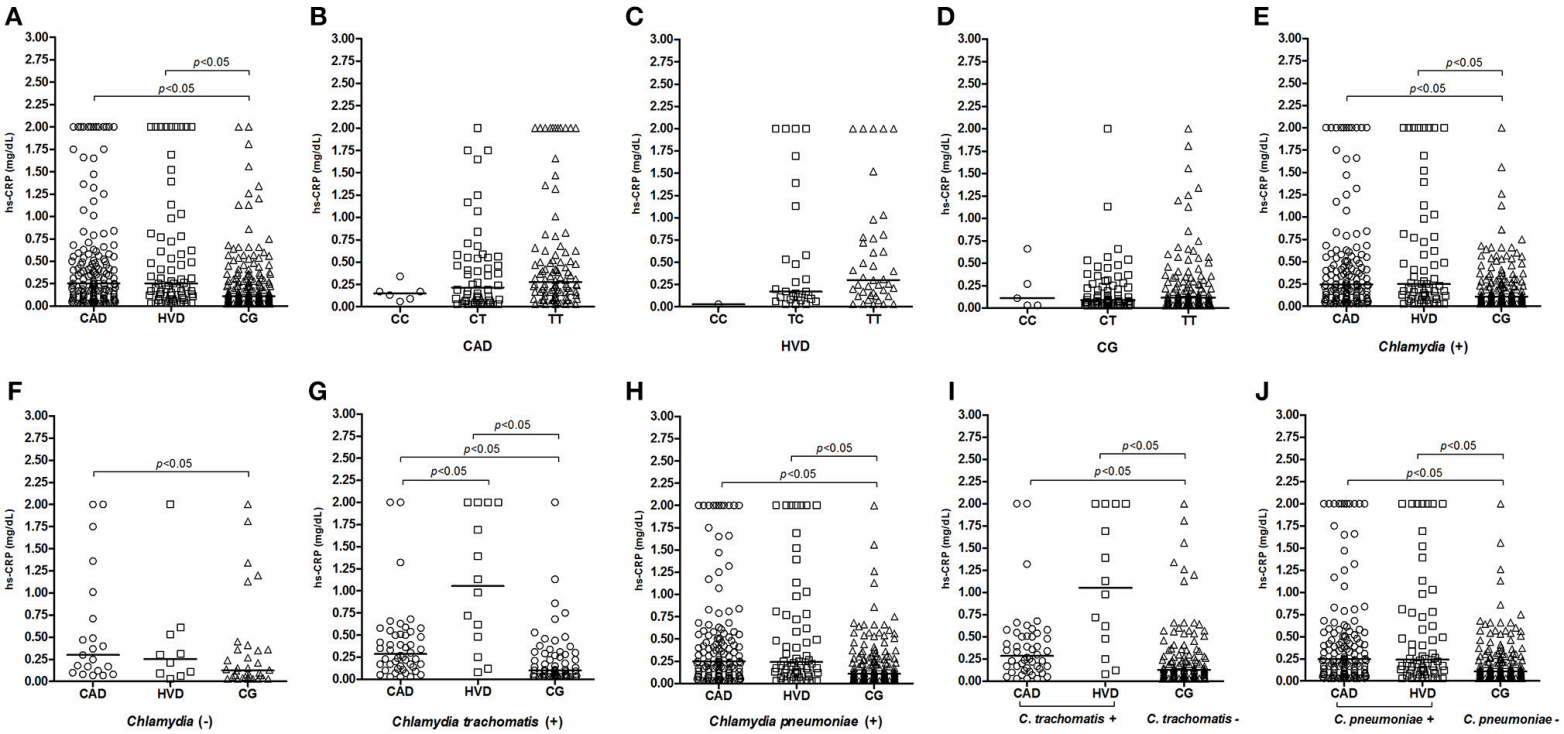
Distribution of plasma C-reactive protein (CRP) levels **(A)** among patients receiving surgical revascularization for coronary artery disease (CAD), patients undergoing valve replacement for heart valve disease (HVD), and control subjects (CG); **(B–D)** according to the different *CRP-*717T>C genotypes; **(E)** among groups serologically positive and **(F)** negative for *Chlamydia*; **(G,I)** among groups with and without previous exposure to *C. trachomatis* and **(H,J)** to *C. pneumoniae*.

**Figure 2:**
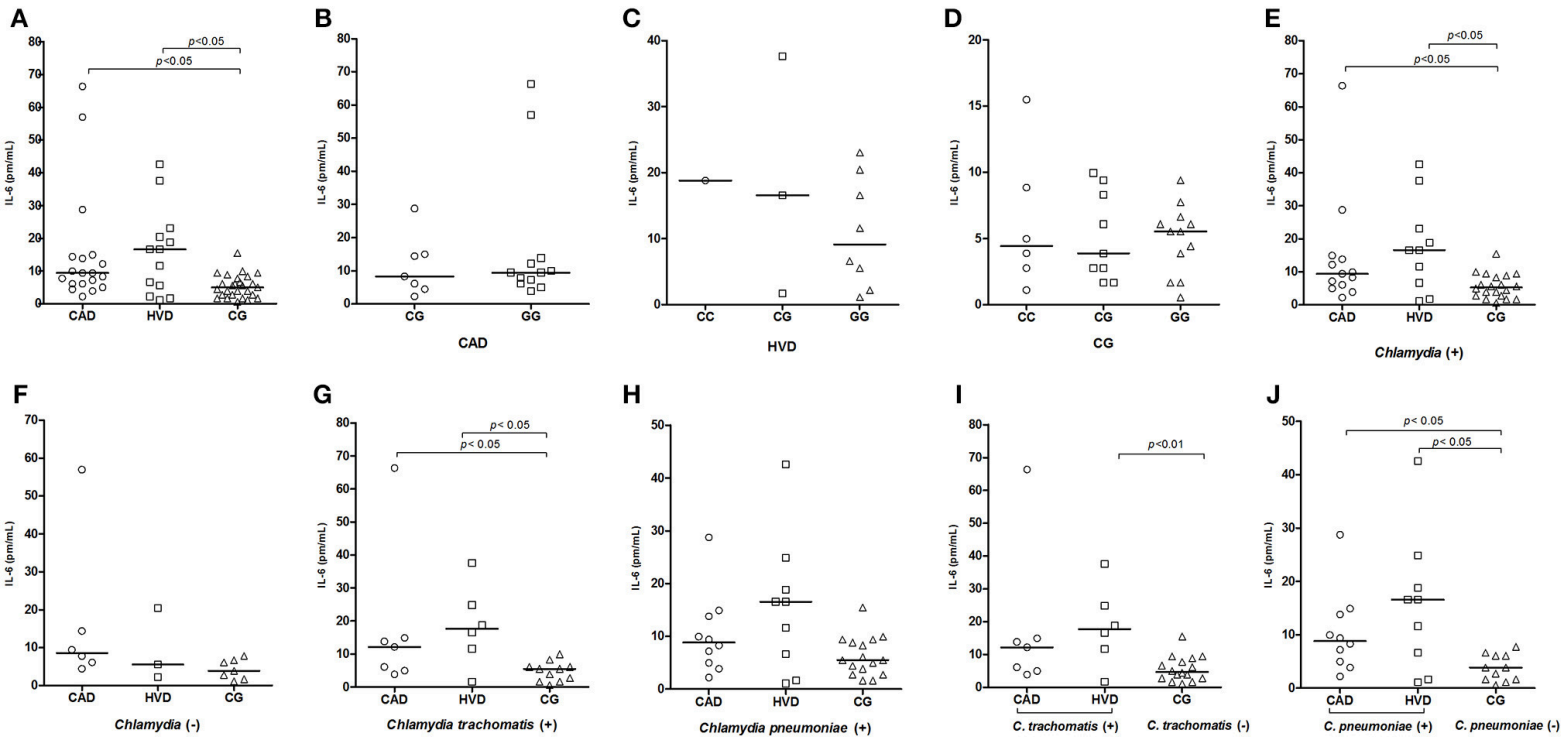
Distribution of plasma IL-6 levels **(A)** among patients receiving surgical revascularization for coronary artery disease (CAD), patients undergoing valve replacement for heart valve disease (HVD) and control subjects (CG); **(B–D)** according to the different *IL-6*-174G>C genotypes; **(E)** among groups serologically positive and **(F)** negative for *Chlamydia*; **(G,I)** among groups with and without previous exposure to *C. trachomatis* and **(H,J)** to *C. pneumoniae*.

**Figure 3:**
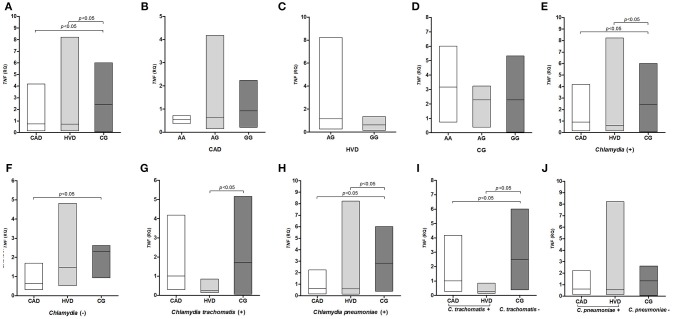
Distribution of *TNF* mRNA levels **(A)** between patients receiving surgical revascularization for coronary artery disease (CAD), patients undergoing valve replacement for heart valve disease (HVD) and control subjects (CG); **(B–D)** according to the different *TNF -*308G>A genotypes; **(E)** among groups serologically positive and **(F)** negative for *Chlamydia*; **(G,I)** among groups with and without previous exposure to *C. trachomatis* and **(H,J)** to *C. pneumoniae*.

**Figure 4:**
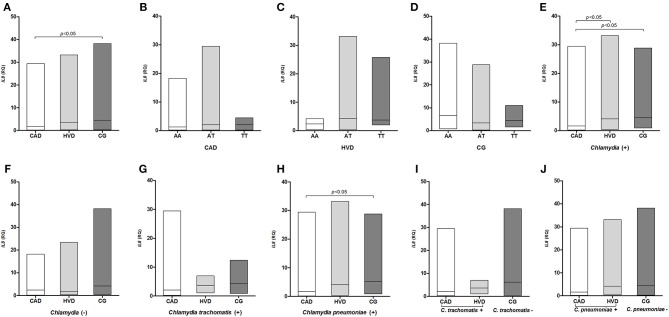
Distribution of the *IL-8* mRNA levels **(A)** among patients receiving surgical revascularization for coronary artery disease (CAD), patients undergoing valve replacement for heart valve disease (HVD) and control subjects (CG); **(B–D)** according to the different *IL8*−251T>A genotypes; **(E)** among groups serologically positive and **(F)** negative for Chlamydia; **(G,I)** among groups with and without previous exposure to *C. trachomatis* and **(H,J)** to *C. pneumoniae*.

The assessment of the plasma levels considered the median values described in Figures 1–4. The CRP values were higher in the CAD and HVD groups than in the controls (Figure 1A). No differences in the plasma CRP levels were found between the different genotypes in each of the investigated groups (Figures 1B–D). The influence of the genotypes on the plasma levels according to previous exposure to *Chlamydia* showed that the values were significantly higher among those already exposed. In the absence of previous contact with the bacterium, the difference was significant only when comparing the CAD and control groups (Figures 1E,F). The comparison of groups according to previous exposure to *C. trachomatis* showed significant differences between the CAD and HVD groups and between these two disease groups and the control group (Figure 1G). When the comparison took into account previous exposure to *C. pneumoniae*, a difference was found only between the patient groups and the control group (Figure 1H). The plasma CRP values among the patients in the CAD and HVD groups previously exposed to *C. trachomatis* or *C. pneumoniae* were significantly higher than the levels in the non-exposed controls (Figures 1I,J). CRP levels were significantly increased among the patient groups as compared with controls, regardless of a previous *Chlamydia* infection.

IL-6 plasma levels were elevated and significantly different in some specific situations, such as when comparing the patient groups (CAD and HVD) with the CG (Figure 2A) and when considering previous exposure to the genus *Chlamydia* and to *C. trachomatis* (Figures 2E,G). Previous exposure to *C. trachomatis* showed a significant difference between the HVD group and the controls (Figure 2I). Previous exposure to *C. pneumoniae* showed a significant difference between both the CAD and HVD groups compared to the controls (Figure 2J). In all comparisons, the expression level was always higher in the HVD group.

### *TNF, IL8*, and *IL10* Gene Expression

The *IL10* mRNA expression level was not significantly different between the patient and control groups when considering the characteristics of genotype, previous exposure to the genus *Chlamydia* or the two *Chlamydia* species (data not shown).

*TNF* expression levels were significantly lower in the patient groups (CAD and HVD) than in the control group (Figure 3A) but were not correlated with the *TNF -*308G>A polymorphism genotypes (Figures 3B–D). The differences between the patient and control groups were evidenced when considering prior exposure to *Chlamydia* (Figure 3E) but remained only between the CAD and controls in persons with no prior exposure (Figure 3F). The *TNF* mRNA levels were significantly decreased in the HVD compared to the control group and according to prior contact with *C. trachomatis* (Figure 3G). However, the decrease was significant for the CAD and HVD groups according to prior contact with *C. pneumoniae* (Figure 3H). The decrease in *TNF* expression was significantly lower when comparing patients in the CAD and HVD groups previously exposed to *C. trachomatis* with the non-exposed controls but not to *C. pneumoniae* (Figures 3I,J).

*IL8* gene expression was also lower among the patient groups than the controls and significantly differed between the CAD and CG groups (Figure 4A), between the CAD and HVD groups, and between the CAD and control groups when considering previous exposure to *Chlamydia* (Figure 4E). The difference between the CAD and CG groups remained significant when previous exposure to *C. pneumoniae* was considered in all three groups (Figure 4H).

## Discussion

Cardiovascular diseases result in the death of ~17.5 million people per year^1^ due to risk factors including hypertension, diabetes, smoking, excess weight, sex, age, ethnicity, genetic inheritance (21, 22), and inflammatory and rheumatic heart disease.

The patients investigated in the present study presented characteristics similar to those of other studies (21, 23, 24). The patients who underwent myocardial revascularization (CAD group) were mostly men with a mean age of 60.4 years and were married. The HVD group, which underwent valve replacement, was represented by a majority of women who were single with an average age of 45.6 years. Although the groups showed differences, the two groups shared a low education level and low family income. These factors do not favor adequate understanding of the protection and care associated with the prevention of heart diseases. Atherosclerosis, which is a disease of multifactorial etiology and valvulopathy caused by congenital disorders or rheumatic fever, may also develop from an infectious component (25).

*Chlamydia pneumoniae* is a bacterium that is frequently involved in the etiology of heart disease and is widespread among adults, who have high prevalence of antibodies (26, 27). The groups involved presented prevalence rates >80%, which was common in other areas of the world (26, 28–32). Since the 1980, evidence of the presence of *C. pneumoniae* in aortic valves has been demonstrated, and the bacterium has been assigned an important role in the calcification process (33). However, failure to detect *C. pneumoniae* is common using methods such as cell culture isolation or nucleic acid detection in cardiovascular tissues, even in populations with a high prevalence of antibodies (24–36). Biopsies of the coronary artery (37), aorta, and atheroma plaque (38) showed the presence of *C. pneumoniae* antigens by immunohistochemistry, which might be relevant in the pathogenesis of atherosclerosis.

Additionally, *C. trachomatis* is a major sexually transmitted bacterium (39) that has an extensive distribution in the Amazon region of Brazil, particularly in Pará (40–42), as has also been observed in the present study among the three groups. The occurrence of cardiopathies by *C. trachomatis* has been demonstrated in experimental studies (43–45) and in case reports (46); however, the role of this bacterium in the pathogenesis of heart disease has rarely been investigated.

Although historical studies on the etiology of atherosclerosis focused on *C. pneumoniae*, our laboratory recently demonstrated (37) the detection of bacterial antigens *in situ* in the aorta and atheroma plaque (*C. pneumoniae*) and in the heart valves (*C. trachomatis*). The present work shows for the first time the amplification of cryptic *C. trachomatis* plasmid DNA in the aorta and mitral valve samples. Two plasma samples corresponding to cases of DNA amplification showed the presence of IgM antibodies against *C. trachomatis*, indicating a recent infection in both patients and demonstrating the entrance of the bacterium in the host and the immediate invasion of tissues of the cardiovascular system. However, in one of these samples, immunohistochemistry did not detect bacterial antigens *in situ* in the aorta, atheroma plaque, or heart valve. Notably, another sample that presented antigens for both species by immunohistochemistry resulted in DNA detection only for *C. trachomatis*. Despite the differences in cellular tropism, *C. pneumoniae, C. trachomatis*, and *C. pecorum* have been associated with endothelial and cardiac muscle infections and have been shown to cause myocarditis in experimental and human models (8, 44, 47).

Although epithelial and muscle cells are not the preferred targets, infected monocytes and macrophages (16) eventually carry *C. trachomatis* to different regions of the human body and are found in cases of endocarditis (48), in the livers of patients with a history of high and prolonged fever (49), and in patients with myocarditis and a history of prostatitis (46). The present study is the first to demonstrate the presence of *C. trachomatis* DNA in cardiovascular tissue, which highlights the possibility that the bacterium may also cause disease. This finding indicates a need for further investigations directed toward elucidating the role of *C. trachomatis* in the pathogenesis of heart disease.

The gene polymorphisms studied were chosen according to their published frequency in the medical literature and the search for their association with different outcomes indicating exposure leading to the infection of an agent or indicating an agent infection leading to disease.

The allelic and genotypic distributions of the polymorphisms investigated in the *CRP, TNF*, and *IL10* genes were not associated with the risk of developing cardiovascular disease even when previous exposure to *C. trachomatis* and *C. pneumoniae* was taken into account. Although the selected polymorphisms had the highest associations with chronic diseases, including atherosclerosis, several other markers (immunological, inflammatory, and biochemical) have been identified and associated with a moderate or high risk for vascular disease (50, 51). Additionally, we should consider the multifactorial condition of diseases that do not depend only on a genetic predisposition but also depend on environmental factors (i.e., a sedentary lifestyle, smoking, and hyperlipidemia) (1).

The results of the present study and other studies showed that the *IL6*−174G>C polymorphism might represent a risk factor for cardiovascular diseases by altering the transcription levels of the gene as well as its plasma levels (52, 53). This polymorphism was initially identified among Caucasians with a G allele frequency of ~0.60 and a C allele frequency of ~0.40 (52). In the present study, higher frequencies of the G allele were found. The allelic distribution is heterogeneous, with the frequency of the G allele in Africans and Asians is similar to the frequency found in the present study, whereas the frequency in Europeans and Americans is lower (54). The differences may be due to the genetic contribution of different ethnic groups (Africans, Amerindians, and Europeans) in the formation of the population of Pará state (55).

Patients with the GG genotype show a two- to three-fold increased risk of developing coronary heart disease and valvulopathy compared to patients with other genotypes (53, 56); additionally, patients with this genotype have a risk of developing different pathologies, including Kaposi's sarcoma, hyperlipidemia, and growth deficiency (Crohn's disease patients) [(57), p. 56]. Heart valve lesions stimulate the inflammatory process and exacerbate the secretion of proinflammatory cytokines, including IL-6 (58). GG genotype was higher than in other studies shown above and is well correlated with the expression and consequent serum levels shown in Figure 2 (discussed below). The major risk of allele G was not clearly detected in the present study.

The GC genotype presented a significantly higher frequency in the control population, indicating possible protection against cardiovascular disease. The presence of the C allele reduces transcriptional activation of the *IL-6* gene and may provide a more balanced cytokine secretion response (52). However, other studies noted that the C allele was associated with higher plasma IL-6 levels (59). Thus, the role of this polymorphism remains controversial (60).

The *IL8*−251A>T polymorphism showed a significantly higher frequency of the AA genotype in the HVD population than in the other groups. This genotype is related to higher transcriptional activity of the gene (61). The heterozygous genotype was found more frequently in the control population than in the patients with valvulopathies, probably because it presented a more balanced profile of IL-8 cytokine production and could act as protection against the disease (62). The role of IL-8 is associated with the inflammatory process characteristic of valvulopathies as well as the secretion of monocyte chemoattractant proteins (MCP-1), adhesion molecules, fibroblast growth factors, and neutrophil chemoattractant (62). In the present study, the AA genotype was shown to be a risk factor for the onset of disease in the HVD group.

The GG genotype for the *IL6*−174G>C polymorphism is apparently a risk factor for the development of cardiovascular disease regardless of whether the serological result indicates previous exposure to genus *Chlamydia* or the two *Chlamydia* species investigated. The GC genotype acts as a protection factor in relation to heart disease. Importantly, previous exposure to *C. trachomatis* is a variable similar to previous exposure to *C. pneumoniae* for the risk of developing heart disease, which has not been clearly demonstrated previously. However, the significance of the *IL8*−251A>T polymorphic genotypes exists only when considering prior exposure to genus *Chlamydia* and the *C. pneumoniae* species. Due to the history of infection by this bacterium, wild genotypes for the *IL6*−174G>C and *IL8*−251A>T polymorphisms may act synergistically and accentuate the mechanisms of the immune response via specific inflammatory stimuli.

The CRP levels were higher in both patient groups than in the controls, indicating that in both cases, an acute inflammatory process was observed, reinforcing the role of CRP as a risk indicator for cardiovascular disease (unstable angina, stable angina, and acute myocardial infarction) and heart valve disease (63, 64). Several polymorphisms in the CRP gene have been shown to be associated with changes in the serum protein concentration, such as the *CRP -*717T>C polymorphism, which appears to influence the serum protein concentration and the development of cardiovascular diseases (65, 66). However, in the present study, there was no evidence of a significant association of the polymorphism with variations in the plasma CRP levels. The TT genotype raised the CRP levels higher than the controls, which was previously demonstrated (67). Despite the importance of the genetic variable, we detected no influence of the isolated form on the CRP levels (66). Previous exposure to *Chlamydia* significantly increased CRP, particularly among those who had never been infected by the bacterium, and only the CAD group showed high levels (similar to those with prior *C. pneumoniae* infection). Previous exposure to *C. trachomatis* significantly increased the levels among the valvulopathy patients. The presence of infection markers for the species can function as a transcriptional stimulus in combination with other factors to increase CRP secretion in valvulopathy patients (68).

The IL-6 levels were also higher in the patient groups, confirming the cytokine's contribution to the disease-related systemic inflammatory process (69, 70). However, no relationship was found between the IL-6 levels and the studied genotypes, which was in contrast to previous studies that found that the GG polymorphism was more frequent in the patient groups (52, 53). The previously demonstrated presence of IL-6 *in situ* is compatible with elevated levels in the two patient groups (37). Importantly, IL-6 played a role in the stimulation of acute phase proteins such as CRP (70), which was also significantly elevated in the patients in the present study.

The high prevalence of *C. pneumoniae* acts as a confounding factor when the results of IL-6 plasma levels are described. The previous exposure to both bacteria was then treated as two independent events, in order to sort out the implication of each bacterium in the inflammatory process. Previous exposure to *Chlamydia* is a variable that maintains the significance of the difference in the IL-6 levels between the patient groups. This factor maintained higher IL-6 levels in the HVD group, whereas the absence of antibodies to *Chlamydia* removed the effect on the increase in the plasma cytokine level. Both *C. trachomatis* and *C. pneumoniae* influence the IL-6 elevation, particularly in the HVD group. The immune response may be exacerbated by increased plasma IL-6 levels (71, 72) in response to microorganisms and other cytokines (73) in addition to the contribution of the effect of CRP on innate immunity and thus the systemic effects of inflammation (74). The exacerbated production of IL-6 is associated with a variety of autoimmune and inflammatory diseases and other conditions, such as atherosclerosis and acute myocardial infarction (68, 69). However, the association of the IL6−174G>C polymorphism with increased IL-6 and CRP levels has been demonstrated, and this polymorphism is considered a risk factor for the development of CAD (53, 75). Moreover, the association of the polymorphism with the persistence of antibodies to *C. pneumoniae* can lead to chronic infection (76).

*TNF, IL8*, and *IL10* gene expression was evaluated. However, no differences were found in the distribution of the IL-10 expression levels in the investigated conditions. IL-10 is an immunosuppressive cytokine that can inhibit the expression of TNF-α, IL-8, and other cytokines even at basal expression levels (77, 78). Immunosuppression was observed in the present study. Both TNF-α and IL-8 were expressed at higher levels in the control subjects than in patients despite the large concentrations detected *in situ* (37) and the lack of an influence by any genotypes of the *TNF* and *IL8* polymorphisms. Previous exposure to *Chlamydia* had a significant influence on the expression levels, but two effects were observed. The decrease in IL-8 expression was more pronounced in the CAD group following exposure to *C. pneumoniae*, whereas reduced *TNF* mRNA levels were associated with prior exposure to *C. trachomatis*.

The demographic characteristics of the patients with coronary artery disease and valvulopathies were similar to the common conditions found in the Brazilian population, and the prevalence of previous infections with *C. trachomatis* and *C. pneumoniae* did not differ from reports concerning Belém and other population groups in the Brazilian Amazon (41, 42). The presence of *C. trachomatis* cryptic plasmid DNA demonstrates the real possibility of new perspectives on the etiology of heart disease.

## Conclusion

The wild genotypes of the *IL6* and *IL8* gene polymorphisms were present at a high frequency and with elevated CRP expression and proved to be good predictors of heart disease. The presence of bacterial antigens *in situ* (37) reinforces the suggestion that *C. trachomatis* can also reach the cardiovascular system and cause disease. The results are relevant to the understanding of genetic susceptibility, the interaction between pro- and anti-inflammatory markers, the control of gene expression, and the role of infectious agents capable of potentiating the inflammatory reaction of heart disease.

## Author Contributions

NA, MI, AV, and RI designed the study. NA, MQ, MA, and IB performed the experiments. NA, MI, SL, RI, AV, and MQ analyzed and interpreted the data. NA, MQ, and RI wrote the manuscript. RI and AV oversaw the experiments and edited the manuscript. NA, MQ, SL, IB, MA, AV, MI, and RI reviewed the manuscript.

### Conflict of Interest Statement

The authors declare that the research was conducted in the absence of any commercial or financial relationships that could be construed as a potential conflict of interest.

## References

[1] World Health Organization Global Atlas on Cardiovascular Disease Prevention Control. Geneva, WHO (2013). Available online at: http://whqlibdoc.who.int/publications/2011/9789241564373_eng.pdf

[2] Galkina E Ley K. Immune and inflammatory mechanisms of atherosclerosis. Annu Rev Immunol. (2009) 27:165–97. 10.1146/annurev.immunol.021908.132620 19302038 PMC2734407

[3] Moreno-Viedma V Amor M Sarabi A Bilban M Staffler G Zeyda M. Common dysregulated pathways in obese adipose tissue and atherosclerosis. Cardiovasc Diabetol. (2016) 15:120. 10.1186/s12933-016-0441-2 27561966 PMC5000404

[4] Pinar A Oç M Akyön Y Farsak B Koçyildirim E Us D. The presence of *Chlamydophila pneumoniae, Helicobacter pylori* and cytomegalovirus in human atherosclerosis detected by molecular and serological methods. Mikrobiyol Bull. (2004) 38:213–22. 15490840

[5] Nazmi A Diez-Roux AV Jenny NS Tsai MY Szklo M Aiello AE. The influence of persistent pathogens on circulating levels of inflammatory markers: a cross-sectional analysis from the Multi-Ethnic Study of Atherosclerosis. BMC Public Health (2010) 10:706. 10.1186/1471-2458-10-706 21083905 PMC2996373

[6] Kuo CC Grayston JT Campbell LA Goo YA Wissler RW Benditt EP. *Chlamydia pneumoniae* (TWAR) in coronary arteries of young adults (15-34 years old). Proc Natl Acad Sci USA. (1995) 92:6911–4. 10.1073/pnas.92.15.6911 7624342 PMC41440

[7] Bayram A Erdogan MB Eksi F Yamak B. Demonstration of *Chlamydophila pneumoniae, Mycoplasma pneumoniae*, Cytomegalovirus, and Epstein-Barr virus in atherosclerotic coronary arteries, nonrheumatic calcific aortic and rheumatic stenotic mitral valves by polymerase chain reaction. Anadolu Kardiyol Derg. (2011) 11:237–43. 10.5152/akd.2011.057 21466993

[8] Gaydos CA Summersgill JT Sahney NN Ramirez JA Quinn TC. Replication of *Chlamydia pneumoniae in vitro* in human macrophages, endothelial cells, and aortic smooth muscle cells. Infect Immun. (1996) 64:1614–20. 8613369 10.1128/iai.64.5.1614-1620.1996 PMC173970

[9] Kuroda S Kobayashi T Ishii N Ikeda J Shinohe Y Houkin K. Role of *Chlamydia pneumoniae*-infected macrophages in atherosclerosis developments of the carotid artery. Neuropathology (2003) 23:1–8. 10.1046/j.1440-1789.2003.00484.x 12722920

[10] Cabbage S Ieronimakis N Preusch M Lee A Ricks J Janebodin K. *Chlamydia pneumoniae* infection of lungs and macrophages indirectly stimulates the phenotypic conversion of smooth muscle cells and mesenchymal stem cells: potential roles in vascular calcification and fibrosis. Pathog and Dis. (2014) 72:61–9. 10.1111/2049-632X.12185 24833344

[11] Kol A Bourcier T Lichtman AH Libby P. Chlamydial and human heat shock protein 60s activate human vascular endothelium, smooth muscle cells, and macrophages. J Clin Invest. (1999) 103:571–7. 10.1172/JCI5310 10021466 PMC408102

[12] Ngeh J Anand V Gupta S. *Chlamydia pneumoniae* and atherosclerosis - what we know and what we don't. Clin Microbiol Infect. (2002) 8:2–13. 10.1046/j.1469-0691.2002.00382.x 11906495

[13] Byrne GI Kalayoglu MV. *Chlamydia pneumoniae* and atherosclerosis: links to the disease process. Am Heart J. (1999) 138:S488–90. 10.1016/S0002-8703(99)70282-6 10539855

[14] Krüll M Kramp J Petrov T Klucken AC Hocke AC Walter C. Differences in cell activation by *Chlamydophila pneumoniae* and *Chlamydia trachomatis* infection in human endothelial cells. Infect Immun. (2004) 72:6615–21. 10.1128/IAI.72.11.6615-6621.2004 15501794 PMC523009

[15] Koehler L Nettelnbreker E Hudson AP Ott N Gérard HC Branigan PJ. Ultrastructural and molecular analyses of the persistence of *Chlamydia trachomatis* (serovar K) in human monocytes. Microb Pathog. (2005) 22:133–42. 10.1006/mpat.1996.0103 9075216

[16] Sun HS Eng EW Jeganathan S Sin AT Patel PC Gracey E. *Chlamydia trachomatis* vacuole maturation in infected macrophages. J Leukoc. (2012) 92:815–27. 10.1189/jlb.0711336 22807527 PMC4050525

[17] Johnston SC Messina LM Browner WS Lawton MT Morris C Dean D. C-Reactive protein levels and viable *Chlamydia pneumoniae* in carotid artery atherosclerosis. Stroke (2001) 32:2748–52. 10.1161/hs1201.099631 11739967

[18] Hajeer AH Hutchinson IV TNF-a gene polymorphism: clinical and biological implications. Microsc Res Tech. (2000) 50:216–28. 10.1002/1097-0029(20000801)50:3<216::AID-JEMT5>3.0.CO;2-Q 10891887

[19] Couper KN Blount DG Riley EM. IL-10: The master regulator of immunity to infection. J Immunol. (2008) 180:5771–7. 10.4049/jimmunol.180.9.5771 18424693

[20] Clarkson TB Estrogen effects on arteries vary with stage of reproductive life and extent of subclinical atherosclerosis progression. Menopause (2007) 4:373–84. 10.1097/GME.0b013e31803c764d 17438515

[21] Safford MM Brown TM Muntner PM Durant RW Glasser S Halanych JH. Association of race and sex with risk of incident acute coronary heart disease events. JAMA (2012) 308:1768–74. 10.1001/jama.2012.14306 23117777 PMC3772637

[22] Clark AM Desmeules M Luo W Duncan AS Wielgosz A. Socioeconomic status and cardiovascular disease: risks and implications for care *Nat*. Rev Cardiol. (2009) 6:712–22. 10.1038/nrcardio.2009.163 19770848

[23] Ruff CT Braunwald E. The evolving epidemiology of acute coronary syndromes. Nat Rev Cardiol. (2011) 8:140–7. 10.1038/nrcardio.2010.199 21173793

[24] Okello E Kakande B Sebatta E Kayima J Kuteesa M Mutatina B. Socioeconomic and environmental risk factors among rheumatic heart disease patients in Uganda. PLoS ONE (2012) 7:e43917. 10.1371/journal.pone.0043917 22952810 PMC3428272

[25] Koh WP Taylor MB Hughes K Chew SK Fong CW Phoon MC. Seroprevalence of IgG antibodies against *Chlamydia pneumoniae* in Chinese, Malays and Asian Indians in Singapore. Int J Epidemiol. (2002) 31:1001–7. 10.1093/ije/31.5.1001 12435775

[26] Burillo A Bouza E Chlamydophila pneumoniae. Infect Dis Clin North Am. (2010) 24:61–71. 10.1016/j.idc.2009.10.002 20171546

[27] Miyashita N Fukano H Yoshida K Niki Y Matsushima T. Seroepidemiology of *Chamydia pneumoniae* in Japan between 1991 and 2000. J Clin Pathol. (2002) 55:115–7. 10.1136/jcp.55.2.115 11865005 PMC1769582

[28] Meza-Junco J Montaño-Loza A Castillo-Martínez L Orea-Tejeda A Remes-Troche JM Villalobos-Zapata I. High prevalence of *Chlamydia pneumoniae* seropositivity in Mexican patients with ischemic heart disease. Arch Med Res. (2004) 35:318–23. 10.1016/j.arcmed.2004.03.005 15325506

[29] Bellido-Casado J Martín-Escudero JC Tasende-Mata J Mena-Martín J Simal-Blanco F Ortiz de Lejarazu R. *Chlamydophila pneumoniae* seroprevalence in adults from the general population. Med Clin. (2006) 126:765–7. 10.1157/13089108 16792979

[30] Agarwal A Chander Y Nagendra A. Serological evidence of chronic Chlamydia pneumonia infection in coronary artery disease. Med J Armed Forces India (2007) 63:229–32. 10.1016/S0377-1237(07)80141-9 27408004 PMC4922735

[31] Atar S Tolstrup K Cercek B Siegel RJ. *Chlamydia pneumoniae* antibody titers and cardiac calcifications: a cross-sectional serological-echocardiographic correlative study. Isr Med Assoc J. (2007) 9:517–20. 17710782

[32] Higuchi-dos-Santos MH Pierri H Higuchi ML Nussbacher A Palomino S Sambiase NV. *Chlamydia pneumoniae* and *Mycoplasma pneumoniae* in calcified nodes of stenosed aortic valves. Arq Bras Cardiol. (2005) 84:443–8. 10.1590/S0066-782X2005000600002 16007307

[33] Andreasen JJ Farholt S Jensen JS. Failure to detect *Chlamydia pneumoniae* in calcific and degenerative arteriosclerotic aortic valves excised during open heart surgery. APMIS (1998) 106:717–20. 10.1111/j.1699-0463.1998.tb00217.x 9740511

[34] Rose AG. Failure to detect *Chlamydia pneumoniae* in senile calcific aortic stenosis or calcified congenital bicuspid aortic valve by immunofluorescence, polymerase chain reaction and electron microscopy. Cardiovasc Pathol. (2002) 11:300–3004. 10.1016/S1054-8807(02)00116-3 12361842

[35] Vainio K Vengen O Hoel T Fremstad H Anestad G. Failure to detect *Chlamydia pneumoniae* in aortic valves and peripheral blood mononuclear cells from patients undergoing aortic valve replacement in Norway. Scand J Infect Dis. (2002) 34:660–3. 10.1080/00365540210147796 12374356

[36] Ferrari M Werner GS Richartz BM Oehme A Straube E Figulla HR. Lack of association between *Chlamydia pneumoniae* serology and endothelial dysfunction of coronary arteries. Cardiovasc Ultrasound (2005) 3:12. 10.1186/1476-7120-3-12 15857519 PMC1097745

[37] Freitas LS Almeida NCC Queiroz MAF Zaninotto MM Fuzi HT Silva AR. *In situ* detection of *Chlamydia pneumoniae*, C. trachomatis, and cytokines among cardiovascular diseased patients from the Amazon region of Brazil. Infect Drug Resist. (2017) 10:109–14. 10.2147/IDR.S123801 28435302 PMC5391863

[38] Zhang L Ishikawa Y Akasaka Y Ito K Gregory S Ishii T. Limited association of *Chlamydia pneumoniae* detection with coronary atherosclerosis. Atherosclerosis (2003) 167:81–8. 10.1016/S0021-9150(02)00383-0 12618271

[39] World Health Organization World Guidelines for the Treatment of Chlamydia trachomatis. Geneva (2016). Available online at: https://www.ncbi.nlm.nih.gov/books/NBK379707/pdf/Bookshelf_NBK379707.pdf 27559553

[40] Ishak MOG Ishak R Cruz AC Santos DE Salgado U. Chlamydial infection in the Amazon region of Brazil. Trans R Soc Trop Med Hyg. (1993) 87:60–2. 10.1016/0035-9203(93)90421-L 8465397

[41] Ishak MOG Ishak R O impacto da infecção por *Chlamydia* em populações indígenas da Amazônia brasileira. Cad de Saúde Pública (2001) 17:385–96. 10.1590/S0102-311X2001000200013 11283769

[42] Ishak MOG Costa MM Almeida NCC Santiago AM Brito WB Azevedo VN. *Chlamydia trachomatis* serotype A infections in the Amazon region of Brazil: prevalence, entry and dissemination. Rev Soc Bras Med Trop. (2015) 48:170–4. 10.1590/0037-8682-0038-2015 25992931

[43] Bachmaier K Neu N De La Maza LM Pal S Hessel A Penninger JM. *Chlamydia* infections and heart disease linked through antigenic mimicry. Science (1999) 283:1335–9. 10.1126/science.283.5406.1335 10037605

[44] Fan Y Wang S Yang X. Chlamydia trachomatis (mouse pneumonitis strain) induces cardiovascular pathology following respiratory tract infection. Infect Immun. (1999) 67:6145–51. 10531278 10.1128/iai.67.11.6145-6151.1999 PMC97004

[45] Mavroveni S Manoussakis M Spargias K Kolovou G Saroglou G Cokkinos DV Myocardial involvement in a patient with chlamydia trachomatis infection. J Card Fail. (2008) 14:351–3. 10.1016/j.cardfail.2008.01.002 18474349

[46] Wang G Burczynski F Hasinoff B Zhong G. Infection of myocytes with chlamydiae. Microbiology (2002) 148:3955–9. 10.1099/00221287-148-12-3955 12480899

[47] Brearley BF Hutchinson DN. Endocarditis associated with *Chlamydia trachomatis* infection. Br Heart J. (1981) 46:220–1. 10.1136/hrt.46.2.220 7272136 PMC482633

[48] Dan M Tyrrell LD Goldsand G. Isolation of *Chlamydia trachomatis* from the liver of a patient with prolonged fever. Gut (1987) 28:1514–6. 10.1136/gut.28.11.1514 3428679 PMC1433683

[49] Karaca E Kayikçioğlu M Onay H Gündüz C Ozkinay F. The effect of interleukin-10 gene promoter polymorphisms on early-onset coronary artery disease. Anadolu Kardiyol Derg. (2011) 11:285–9. 10.5152/akd.2011.077 21543297

[50] Nair J Shanker J Jambunathan S Arvind P Kakkar VV. Expression analysis of leukotriene-inflammatory gene interaction network in patients with coronary artery disease. J Atheroscler Thromb. (2014) 21:329–45. 10.5551/jat.20123 24366255

[51] Fishman D Faulds G Jeffery R Mohamed-Ali V Yudkin JS Humphries S. The effect of novel polymorphisms in the interleukin-6 (IL-6) gene on IL-6 transcription and plasma IL-6 levels, and an association with systemic-onset juvenile chronic arthritis. J Clin Invest. (1998) 102:1369–76. 10.1172/JCI2629 9769329 PMC508984

[52] Giacconi R Cipriano C Albanese F Boccoli G Saba V Olivieri F. The−174G/C polymorphism of IL-6 is useful to screen old subjects at risk for atherosclerosis or to reach successful ageing. Exp Gerontol. (2004) 39:621–8. 10.1016/j.exger.2003.12.013 15050298

[53] Antonicelli R Olivieri F Bonafe M Cavallone L Spazzafumo L Marchegiani F. The interleukin-6−174 G > C promoter polymorphism is associated with an higher risk of death after an acute coronary syndrome in male elderly patients. Int J Cardiol. (2005) 103:266–71. 10.1016/j.ijcard.2004.08.064 16098388

[54] Santos NP Ribeiro-Rodrigues EM Ribeiro-dos-Santos AK Pereira R Gusmão L Amorim A. Assessing individual interethnic admixture and population substructure using a 48-insertion-deletion (INSEL) ancestry-informative marker (AIM) panel. Hum Mutat. (2010) 31:184–90. 10.1002/humu.21159 19953531

[55] Myśliwska J Wieckiewicz J Hak L Siebert J Rogowski J Szyndler K. Interleukin 6 polymorphism corresponds to the number of severely stenosed coronary arteries. Eur Cytokine Netw. (2006) 17:181–8. 10.1684/ecn.2006.0035 17194638

[56] Fernandez-Real JM Broch M Vendrell J Richart C Ricart W. Interleukin-6 gene polymorphism and lipid abnormalities in healthy subjects. J Clin Endocrinol Metab. (2000) 85:1334–9. 10.1210/jcem.85.3.6555 10720087

[57] Sawczenko A Azooz O Paraszczuk J Idestrom M Croft NM Savage MO. Intestinal inflammation-induced growth retardation acts through IL-6 in rats and depends on the -174 IL-6 G/C polymorphism in children. Proc Natl Acad Sci USA. (2005) 102:13260–5. 10.1073/pnas.0503589102 16150725 PMC1198995

[58] Niu W Liu Y Qi Y Wu Z Zhu D Jin W. Association of interleukin-6 circulating levels with coronary artery disease: a meta-analysis implementing mendelian randomization approach. Int J Cardiol. (2012) 157:243–52. 10.1016/j.ijcard.2011.12.098 22261689

[59] Yin YW Li JC Zhang M Wang JZ Li BH Liu Y. Influence of interleukin-6 gene−174G>C polymorphism on development of atherosclerosis: a meta-analysis of 50 studies involving 33,514 subjects. Gene (2013) 529:94–103. 23954871 10.1016/j.gene.2013.07.074

[60] Wang N Zhou R Wang C Guo X Chen Z Yang S. −251 T/A polymorphism of the interleukin-8 gene and cancer risk: a HuGE review and meta-analysis based on 42 case-control studies. Mol Biol Rep. (2012) 39:831–41. 10.1007/s11033-011-1042-5 21681427

[61] Harada A Sekido N Akahoshi T Wada T Mukaida N Matsushima K. Essential involvement of interleukin-8 (IL-8) in acute inflammation. J Leukoc Biol. (1994) 56:559–64. 10.1002/jlb.56.5.559 7964163

[62] Ridker PM Rifai N Rose L Buring JE Cook NR. Comparison of C-reactive protein and low-density lipoprotein cholesterol levels in the prediction of first cardiovascular events. N Engl J Med. (2002) 347:1557–65. 10.1056/NEJMoa021993 12432042

[63] Sinning JM Bickel C Messow CM Schnabel R Lubos E Rupprecht HJ. Impact of C-reactive protein and fibrinogen on cardiovascular prognosis in patients with stable angina pectoris: the AtheroGene study. Eur Heart J. (2006) 27:2962–8. 10.1093/eurheartj/ehl362 17132649

[64] Brull DJ Serrano N Zito F Jones L Montgomery HE Rumley A. Human CRP gene polymorphism influences CRP levels: implications for the prediction and pathogenesis of coronary heart disease. Arterioscler Thromb Vasc Biol. (2003) 23:2063–9. 10.1161/01.ATV.0000084640.21712.9C 12842840

[65] Hage FG Szalai AJ. C-reactive protein gene polymorphisms, C-reactive protein blood levels, and cardiovascular disease risk. J Am Coll Cardiol. (2007) 50:1115–22. 10.1016/j.jacc.2007.06.012 17868801

[66] Wang Q Ding H Tang Jr Zhang L Xu YJ Yan JT. C-reactive protein polymorphisms and genetic susceptibility to ischemic stroke and hemorrhagic stroke in the Chinese Han population. Acta Pharmacol Sin. (2009) 30:291–8. 10.1038/aps.2009.14 19262552 PMC4002404

[67] Skowasch D Tuleta I Steinmetz M Pabst S Preusse CJ Welz A. Pathogen burden in degenerative aortic valves is associated with inflammatory and immune reactions. J Heart Valve Dis. (2009) 18:411–7. 19852145

[68] Abeywardena MY Leifert WR Warnes KE Varghese JN Head RJ. Cardiovascular biology of interleukin-6. Curr Pharm Des. (2009) 15:1809–21. 10.2174/138161209788186290 19442192

[69] Vakili H Ghaderian SM Akbarzadeh Najar R Tabatabaei Panah AS Azargashb E. Genetic polymorphism of interleukin-6 gene and susceptibility to acute myocardial infarction. Coron Artery Dis. (2011) 22:299–305. 10.1097/MCA.0b013e328346b848 21512395

[70] Castell JV Gómez-Lechón MJ David M Fabra R Trullenque R Heinrich PC. Acute-phase response of human hepatocytes: regulation of acute-phase protein synthesis by interleukin-6. Hepatology (1990) 12:1179–86. 10.1002/hep.1840120517 1699862

[71] Mazzone A Epistolato MC De Caterina R Storti S Vittorini S Sbrana S. Neoangiogenesis, T-lymphocyte infiltration, and heat shock protein-60 are biological hallmarks of an immunomediated inflammatory process in end-stage calcified aortic valve stenosis. J Am Coll Cardiol. (2004) 43:1670–6. 10.1016/j.jacc.2003.12.041 15120829

[72] Vats V Agrawal T Salhan S Mittal A. Primary and secondary immune responses of mucosal and peripheral lymphocytes during *Chlamydia trachomatis* infection. FEMS Immunol Med Microbiol. (2007) 49:280–7. 10.1111/j.1574-695X.2006.00196.x 17328762

[73] Kishimoto T. Interleukin-6: from basic science to medicine - 40 years in immunology. Annu Rev Immunol. (2005) 23:1–21. 10.1146/annurev.immunol.23.021704.115806 15771564

[74] Brennan FM Mclnnes IB. Evidence that cytokines play a role in rheumatoid arthritis. J Clin Invest. (2008) 118:3537–45. 10.1172/JCI36389 18982160 PMC2575731

[75] Satti HS Hussain S Javed Q. Association of Interleukin-6 gene promoter polymorphism with coronary artery disease in Pakistani families. Sci World J. (2013) 2013:538365. 10.1155/2013/538365 24363620 PMC3865736

[76] Rantala A Lajunen T Juvonen R Paldanius M Silvennoinen-Kassinen S Peitso A. Interleukin-6-174 G/C promoter polymorphism is associated with persistence of Chlamydia pneumoniae antibodies in young men. Scand J Immunol. (2011) 74:95–9. 10.1111/j.1365-3083.2011.02542.x 21352255

[77] Rajasingh J Bord E Luedemann C Asai J Hamada H Thorne T. IL-10-induced TNF-alpha mRNA destabilization is mediated via IL-10 suppression of p38 MAP kinase activation and inhibition of HuR expression. FASEB J. (2006) 20:2112–4. 10.1096/fj.06-6084fje 16935932

[78] Yilma AN Singh SR Fairley SJ Taha MA Dennis VA. The anti-inflammatory cytokine, interleukin-10, inhibits inflammatory mediators in human epithelial cells and mouse macrophages exposed to live and UV-inactivated *Chlamydia trachomatis*. Mediat Inflamm. (2012) 2012:520174. 10.1155/2012/520174 22529524 PMC3317056

